# The Intangible Magic of Celebrity Marketing

**DOI:** 10.1371/journal.pmed.0010042

**Published:** 2004-11-30

**Authors:** Ray Moynihan

## Abstract

Drug industry insiders share their tips on using celebrities to market drugs and diseases

## Teaser

Drug industry insiders share their tips on using celebrities to expand markets

As the Hispanic world well knows, the word in Spanish for advertising is ‘propaganda’, its meaning derived literally from the propagation of the faith, the antithesis of science's Enlightenment ideals. The old word somehow seems perfect for describing the new world of drug promotion and its growing use of the famous face. Like the catholic cardinals of the 17th century, many of the feted celebrities of the 21st are now engaged in spreading the word. Now, as then, the religion promises miraculous breakthroughs, wonder cures, and sometimes even eternal life. The difference is that this time around, the stars are earning fat fees from the marketing departments of giant pharmaceutical companies. And if the latest revelations from industry insiders are anything to go by, their hefty investments in celebrity selling are well worth it.

## Celebrity Selling

The epicentre of this phenomenon is of course the United States, where companies routinely hire celebrities to attract attention to the latest drugs and the diseases that go with them. Pfizer famously paid presidential hopeful Bob Dole to promote awareness of erectile dysfunction as sildenafil (Viagra) was hitting the market. Wyeth hired supermodel Lauren Hutton to hawk hormone replacement therapy and menopause. GSK contracted football star Ricky Williams to sell social anxiety disorder, helping make paroxetine (Paxil)—briefly—the world's top-selling antidepressant. Even the dead are raising awareness, with the estate of Errol Flynn now enlisted to help promote cardiovascular disease as a household name [1]. The celebrity, living or dead, becomes integral to a drug marketing strategy that includes paid advertising and aggressive public relations campaigns that can produce media appearances on the likes of *Oprah* and *The Today Show*. According to celebrity brokers, the star's remuneration package, though always confidential, can range from $20,000 to $2 million.[Fig pmed-0010042-g001]

**Figure pmed-0010042-g001:**
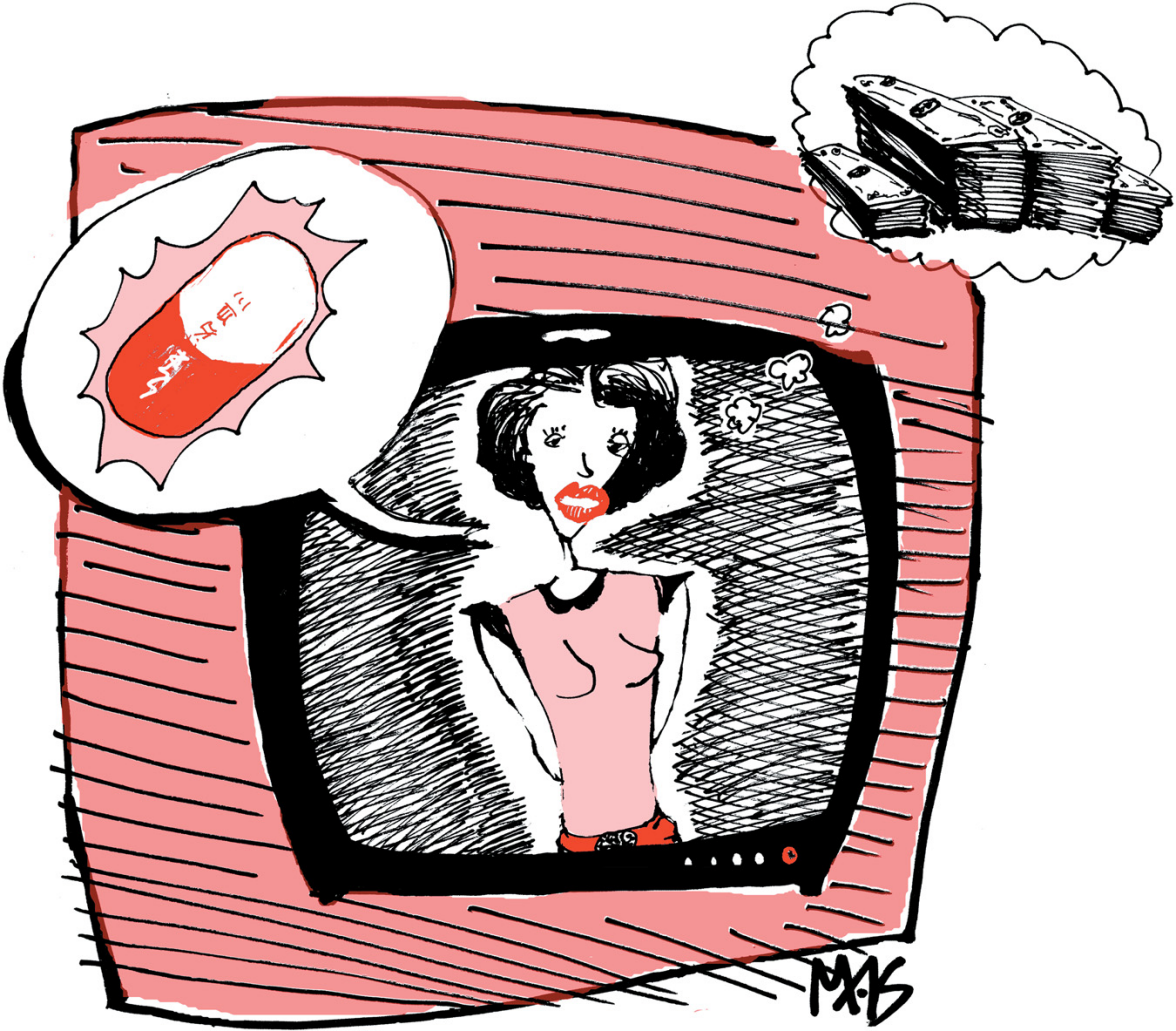
Celebrities often hide their conflicts of interest (Illustration: Margaret Shear)

‘A partnership between a celebrity and a brand has an intangible sort of magic’, writes a senior marketing executive at Amgen, in an extremely candid piece published recently in an industry trade magazine [2]. Amgen is the Californian biotech firm that hired handsome ‘West Wing’ star Rob Lowe to help market an anti-infection drug. Lowe was reportedly paid more than $1 million by Amgen, though there is speculation that part of the fee might flow to charity [3]. In her report, Amgen's Osnat Benshoshan shares some thoughtful tips with her peers among the pharmaceutical marketing fraternity: ‘use an A-list celebrity’; find a ‘news-hook’ that links the celebrity and your product; develop some simple messages; and make sure the celebrity delivers them at every appearance.

Benshoshan then reveals why on-air talk-show appearances on ‘top-tier media venues’ like *The Rosie Show* can be better forums for celebrities than straight advertisements, which are governed by regulations. ‘The great advantage over advertising is that the airtime is practically free, and there is no fair balance to worry about’ she writes [2]. The downside with a media interview, she laments, is that compared to a scripted ad, ‘the situation is less controllable. It can be tricky for the celebrity to ensure that all product messages are delivered….’ Her other big tip for drug-makers is to rate your prospective celebrity with a ‘Q score’, a measure of their likeability and recognisability with the public. Apparently Rob Lowe's Q score was high with women over fifty, a key target of the Amgen campaign [3].

Another recent report from within the industry draws on public opinion survey data to guide drug company marketers on the selection and ‘effective use’ of celebrity spokespersons [4]. The survey was conducted by a Seattle firm called NexCura Inc., in partnership with the trade magazine that published the study. The major findings echo the insights of the Amgen executive about credibility, and underline the importance of your star being perceived as generally trustworthy, and specifically knowledgeable about the condition on which they are hired to speak. Perhaps not surprisingly, the survey found that people diagnosed as suffering chronic conditions were far more attentive to celebrity messages on health than the general public.

The issue of credibility is important, the NexCura Inc. researchers point out, because ‘the credibility rating is used as a surrogate for “buying” behavior’—an intermediate measure of whether the star can persuade people to request the target drug from their doctor. The survey found that Bob Dole was still the most recognisable celebrity marketer with the United States public, but that the skater Dorothy Hamill—currently promoting Merck's arthritis medication rofecoxib (Vioxx)—took the lead in the credibility stakes. Significantly though, almost three-quarters of those surveyed were correctly able to identify Bob Dole with Pfizer's Viagra, despite the fact that the advertisements in which he appeared were ‘unbranded’ ads for erectile dysfunction. The researchers concluded by recommending that drug companies choose a celebrity with personal experience of the target condition; choose someone trustworthy—perhaps a newsreader or sports figure; and choose someone who will promote a single cause or brand rather than multiple ones.

Ironically, the NexCura survey also found two-thirds of medical consumers agreed with the proposition that celebrities were ‘just doing it for the money and can't be trusted’.

## The Trouble with Celebrity Selling

The first problem here is that the public is often not even informed whether a celebrity is receiving money from a drug company. In the case of TV star Rob Lowe, there was no mandated requirement for him to disclose his link with Amgen when appearing on media shows watched by millions. According to one industry insider familiar with the case, who did not want to be named, ‘it depended if he remembered to say it, and whether he was asked’. The media's failure to disclose relevant conflicts of interest when covering healthcare is well established [5]. When *Frasier* star Kelsey Grammer and his wife were promoting irritable bowel syndrome on top-rating TV shows, viewers thought the pair were speaking on behalf of an independent foundation. In fact the couple's fee had flowed from GSK, which was at that time preparing the market for alosetron (Lotronex), a controversial new drug that carried modest benefits and severe side effects, including possible death [6].

Equally as serious is the lack of any formal requirement for stars or media outlets to spell out drug side effects along with benefits when celebrities are pushing products or conditions. Lauren Hutton can be quoted, in magazine articles read by millions of readers, as saying, ‘My No. 1 secret is estrogen’ without any need for her, or the magazine, to list the dangers of the hormone replacement therapy made by her sponsor [7]. But perhaps most troubling is the way celebrities, with their star power, can help to fundamentally shift the public debate about major health problems.

While Prince Charles's companion Camilla Parker Bowles takes no money from drug companies, she did choose to make an important public statement about the bone condition osteoporosis at an international conference funded by Lilly, a company promoting a medication for the condition [8]. Camilla's call for early intervention and greater use of expensive tests and technologies for the primary prevention of osteoporosis drew on materials sponsored by the pharmaceutical industry, and was synchronised with simplistic industry marketing messages. Camilla's high-profile intervention at a drug company sponsored forum, albeit unwittingly, helps keep the focus on biochemical causes of, and biochemical solutions to, the much wider public health problem of fractures. Moreover these simple marketing messages undermine the complexity of the cost-effectiveness arguments that are central to any rational debate about the equitable distribution of health care resources. Other high-profile figures attending the same conference eagerly accepted Lilly money, and one, former Texas Governor Ann Richards, blatantly promoted Lilly's drug during an interview on CNN's Larry King Show just days later [8].

## The Future of Celebrity Selling

With pharmaceutical marketing, it is clear that nothing short of a Vatican II-style reform is required, though there are already encouraging signs of change. Scientific journals are slowly disentangling themselves from unhealthy industry influence over what they publish, and public access to clinical trial data is daily a closer reality [9]. However, a less distorted scientific record about healthcare products is meaningless without regulations on how important science is communicated to the public. Celebrities paid by drug companies to promote drugs, or ‘raise awareness’ about disease, should be subject to the same rules as direct-to-consumer advertising, which would mean prohibition in many nations and much more fulsome disclosure in the United States than is currently the case. At the very least, public disclosure of a product's risks and benefits, and the magnitude of the celebrity's fee, should be mandatory and routine. Let's see what that does to their Q rating.
